# Barnaculate Carcinoma in Four Patients: Verrucoid Squamous Cell Carcinoma Subtype with *TERT* and *HRAS* Oncogenic Variants

**DOI:** 10.1007/s12105-025-01870-3

**Published:** 2026-01-05

**Authors:** Yu Liang, Michelle Afkhami, Lester D. R. Thompson, Hanlin Gao, Ellie G. Maghami, Massimo D’Apuzzo, Sue Chang, Diana Bell, Mihan Telatar, Thomas J. Gernon, Huiqing Wu

**Affiliations:** 1 Department of Pathology, City of Hope National Medical Center, 1500 E. Duarte Rd, Duarte, CA USA; 2 Head and Neck Pathology Consultations, Woodland Hills, CA USA; 3 Fulgent Genetics, El Monte, CA USA; 4 Department of Surgery, City of Hope National Medical Center, Duarte, CA USA; 5 Present Address: Department of Pathology and Otolaryngology, University of Pittsburgh Medical Center, Pittsburgh, PA USA

**Keywords:** Squamous cell carcinoma, Barnaculate carcinoma, Proliferative verrucous leukoplakia, *TERT* promoter mutation/oncogenic variant, *HRAS* mutation/oncogenic variant

## Abstract

Well-differentiated squamous cell carcinoma (SCC) in the oral cavity often has limited pleomorphism. Commonly seen in patients with proliferative verrucous leukoplakia, barnaculate carcinoma (BC) is a newly recognized, distinct subtype of well-differentiated SCC which presents with obvious architecture abnormalities but lacks significant pleomorphism. As such, reaching this diagnosis can be extremely challenging. In this study, molecular analysis of six BC specimens from four different patients demonstrated recurrent alterations in telomerase reverse transcriptase (*TERT*) promoter region and Harvey rat sarcoma virus (*HRAS*) gene. The identification of these oncogenic variants may be a useful adjunct to reaching the diagnosis.

**Supplementary Information:**

The online version contains supplementary material available at 10.1007/s12105-025-01870-3.

## Introduction

Squamous cell carcinoma (SCC) is the most common type of cancer affecting the lip and oral cavity. Oral SCC accounts for over 300,000 new annual cases world-wide, and 50% of these patients die from disease within five years [1, 2]. The common risk factors are excess alcohol consumption, tobacco use (smoking, snuff, and/or chewing), and betel nut chewing. Other risk factors include immune suppression and certain genetic tumor syndromes [3]. Surgeries with adequate resection margins, together with chemotherapy and radiation therapy, remain the main treatments. However, the overall prognosis is poor due to the late initial presentation and diagnosis at advanced tumor stage. Thus, early detection and accurate pathologic diagnosis play critical roles in the management of this cancer.

Oral SCC is a group of morphologically, biologically, and clinically heterogenous and diverse malignant tumors. The histologic diagnosis of oral SCC and grading of precursor dysplastic lesions rely on the identification of pleomorphism, architectural disorganization, dysmaturation, abnormal keratinization, and often the presence of atypical mitoses. However, several subtypes of oral SCCs show distinct morphologic features. Well-differentiated SCC in the oral cavity often has limited pleomorphism with subtle architectural disorder. Verrucous carcinoma (VC) is well recognized as a unique carcinoma with well-differentiated exophytic and endophytic squamous epithelial proliferation creating a broad-pushing border of infiltration below the adjacent epithelium, church spire-type hyperkeratosis, parakeratin-filled crypts and clefts, broad rete, and without significant cytologic atypia [4, 5]. Biopsy of sufficient depth and size are required to accurately diagnose this subtype of SCC. A newly recognized distinct subtype of well-differentiated SCC in patients with proliferative verrucous leukoplakia (PVL) has been referred to as barnaculate carcinoma (BC) [5]. As proposed and described by Thompson, et al., this lesion is characterized by notable bulky epithelial proliferation with voluminous acanthotic, exophytic, and endophytic-appearing columns that exhibit complex foldings and invaginations that do not meaningfully extend below the level of the adjacent uninvolved epithelium. Church spire-type hyperkeratosis is generally absent, although keratosis is commonly present. Specifically, this subtype of SCC presents with obvious architecture abnormalities but lacks significant cytological pleomorphism. The bulky, endo- and exophytic growth frequently lacks destructive stromal invasion, but the sheer volume of the epithelial proliferation is what aids in making the diagnosis. As such, the diagnosis of this subtype of SCC presents a diagnostic challenge when evaluating samples from oral mucosal lesions that are superficial or limited, and is confounded by both inter- and intra-observer variability, especially when differentiating “reactive” squamous proliferations from precursor lesions and even from some conventional subtypes of SCC.

The mechanism of transition from benign squamous mucosa to a precursor lesion, and subsequently to SCC is still poorly understood [2]. Various genetic alterations, such as gene pathogenic variants and genomic instability have been described in head and neck SCC (HNSCC) [6]. These include genetic alterations in *TP53*, *PIK3CA*, *NOTCH1*, *KMT2D*, etc. Studies have also shown alterations in signaling pathways involving the Erb family of receptor tyrosine kinases of ErbB-1 (EGFR), ErbB-2 (Her-2/neu), ErbB-3 (Her3) and ErbB-4 (Her4), as well as MAPK, WNT and PI3K/AKT/mTOR signaling [7]. Malignant tumor cells usually have unlimited proliferation; one way to achieve this state is by telomerase activation [6, 8]. Telomerase activation is a hallmark of malignant transformation and is commonly detected in malignant neoplasms. The upregulation of telomerase involves genetic and epigenetic alterations of telomerase reverse transcriptase (*TERT*) gene, such as amplification, gene rearrangement, and promoter alterations [9]. *TERT* promoter oncogenic variants have been well documented in human cancers, including HNSCC. In a study performed by Boscolo-Rizzo, et al. [10], 12% of the HNSCCs had *TERT* promoter oncogenic variants and a notable 37% of oral SCC carried *TERT* promoter oncogenic variants. In a different study by Yilmaz, et al. [6], a substantial 75% of oral SCC had *TERT* promoter variants.

Rat sarcoma virus (RAS) family proteins are important players in signal transduction pathways controlling cell proliferation, differentiation, and survival [11]. There are three members in the RAS family, *HRAS*, *KRAS*, and *NRAS,* with significant sequence homology and functional overlap. It is estimated that about 19% of cancer patients carry *RAS* oncogenic variants. Among all three *RAS* homologs, *HRAS* has the lowest overall alteration frequency (1.3%) when all types of cancers are considered. However, for HNSCC, *HRAS* has the highest mutation rate (5.1%) when compared to *KRAS* (2.0%) and *NRAS* (1.6%) [12]. Another study has reported that among the total of 193 patients from 4 datasets, *HRAS* oncogenic variants have been found in 3.3% of HPV-negative HNSCCs, in which 60% of patients co-occurred with *TERT* variants [13].

In the current study, we report the clinicopathologic and molecular findings of six BC specimens from four different patients. The BC characteristic *TERT* and *HRAS* oncogenic variants may provide a valuable diagnostic adjunct when evaluating this subtype of SCC affecting the oral cavity.

## Materials and Methods

## Patient Selection

All six available specimens with the diagnosis of BC and two cases of conventional SCC (CSCC) were identified within the archives of the Department of Pathology, City of Hope National Medical Center (COHNMC), Duarte, CA, USA. The study was approved by the Institutional Review Board (IRB) of COHNMC. All patients had clinical and pathological material for evaluation, with appropriate clinical follow-up. An additional three PVL cases without any known cancer diagnosis were evaluated as a control group.

## Histologic Review

Materials were reviewed for inclusion using published criteria for consensus diagnosis [5]. All cases met inclusion criteria as evaluated by LDRT, YL, and HW.

## Molecular Analysis

Molecular analysis of specimens was performed in different CLIA laboratories (Table 2). The status of *TERT* gene alterations for BCs from patients 1–4, CSCCs from patients 2, 5 and 6, reactive squamous mucosa from patient 3, and non-cancerous PVL from patient 7 was analyzed by Sanger sequencing at Clinical Molecular Diagnostic Laboratory (CMDL) of COHNMC, Duarte, CA, USA. The status of *HRAS* gene alterations for BCs and CSCCs was analyzed by HopeSeq Solid Tumor Panel (up to 523 genes) at CMDL of COHNMC, Duarte, CA, USA; for CSCC from patient 2 was analyzed by The Lumera NGS Profile of Solid Tumor (523 genes) at Fulgent Genetics, El Monte, CA, USA; the non-cancerous lesion within PVL from patient 7 was analyzed by RAS Pathway Panel (*HRAS*, *KRAS* and *NRAS* hotspot panel by next-generation sequencing on the Thermofisher platform) at CMDL of COHNMC, Duarte, CA, USA. The status of both *TERT* and *HRAS* gene alterations for BC from patient 2 (Specimen 2C, see Table 2) was analyzed by GEM Extra® (19,396 genes [14]) at Ashion Analytics, Phoenix, AZ, USA.

## Result

## Clinical Information

The relevant clinical information is summarized after review of patient’s medical record and/or discussion with the treating clinicians (Table 1). There were two female and two male patients with BC, all with clinical features of PVL. The age range at first BC diagnosis was 64–71 years. Multifocal, previous leukoplakia lesions were documented.

**Table 1:** Clinical history of patients with Barnaculate Carcinoma

Patient	Sex	Social history	Age at 1st BC event	Disease-related history in oral cavity
1	Female	Ex-smoker	71 years old	1. Multiple reported papillomatous or/dysplastic squamous lesions in gingiva, mandibular buccal sulcus and alveolus since about 3 years prior to the first diagnosis of *BC*^§^ 2. First diagnosis of *BC* in anterior floor of mouth, gingiva, and right lower molar below tooth#30 3. BC* (1A) in right lower gum 8 months after the first diagnosis of *BC* 4. CSCC in bilateral lower gum, right lower prevelar area, behind tooth#24, and left lower molar region 14–15 months after the first diagnosis of *BC* 5. BC* (1B) in gingiva adjacent to tooth #31 27 months after the first diagnosis of *BC*
2	Male	Ex-smoker	69 years old	1. Reported history of bilateral oral carcinomatosis 20 years prior to the first diagnosis of *BC*^§^ 2. Invasive CSCC in left tongue 8 years prior to the first diagnosis of *BC* 3. First diagnosis of BC* (2A) with superficial invasion in right tongue 4. CSCC* (2B) in left lower gum, left anterior oral cavity, midline alveolar and anterior bone 14–17 months after the first diagnosis of *BC* 5. Reactive lesions in gum and soft palate within a period of 25–46 months after the first diagnosis of *BC* 6. BC* (2C) in left upper lip, labial vestibule and anterior gum 49 months after the first diagnosis of *BC* 7. *BC* in left upper gum 51 months after the first diagnosis of *BC*
3	Female	Non-smoker	64 years old	1. First diagnosis of *BC* with superficial invasion in right dorsal lateral tongue 2. Reactive squamous mucosa in right tongue within a period of 5–36 months after the first diagnosis of *BC* 3. BC* (3A) with superficial invasion in right partial tongue 43 months after the first diagnosis of *BC* 4. Inflammatory squamous lesion in right upper gum 56 months after the first diagnosis of *BC*
4	Male	Ex-tobacco chewer/Non-smoker	65 years old	1. *BC* in left buccal mucosa 2. BC* (4A) in right lower lip 36 months after the first diagnosis of *BC* 3. Candida infection and atypical verruciform squamoproliferative lesion in right buccal mucosa with a period of 37–46 months after the first diagnosis of *BC*

*BC* barnaculate carcinoma, *CSCC* conventional squamous cell carcinoma

^*^: specimens with molecular analysis followed by specimen codes in parentheses;^§^: case(s) not available for re-review

### Patient 1

At age of 71, a female former smoker was first diagnosed with BC in the right of midline anterior floor of mouth, gingiva, and right lower molar below tooth #30. Within 3 years before this BC diagnosis, the patient had multiple reported papillomatous or/dysplastic squamous lesions, affecting the gingiva, mandibular buccal sulcus, and alveolus. She developed her second event of BC 8 months later in the right lower gum, and the third event 27 months later in the gingiva adjacent to tooth#31, respectively. Between the second and third events of BC, she also presented with keratinizing well-differentiated CSCC.

### Patient 2

A male former smoker was diagnosed with BC in the anterior right tongue at 69 years of age. The patient had his second BC event 49 months later in the left upper lip, labial vestibule and anterior gum, and the third BC event 51 months later in the left upper gum, respectively. He also had a reported history of bilateral oral carcinomatosis 20 years ago, and well-differentiated CSCC affecting the left tongue 8 years ago. Further, between the first and second BC events, he developed well-differentiated CSCC in the left lower gum, left anterior oral cavity, and midline alveolus.

### Patient 3

A female non-smoker was diagnosed with BC at 64 years of age, affecting the right dorsal lateral tongue. The BC recurred 43 months later in the right lateral tongue.

### Patient 4

A 65-year-old male patient, former tobacco chewer, presented with left buccal mucosal BC. He developed another BC about 36 months later in the right lower lip.

## Pathology Examination

**Fig. 1:**
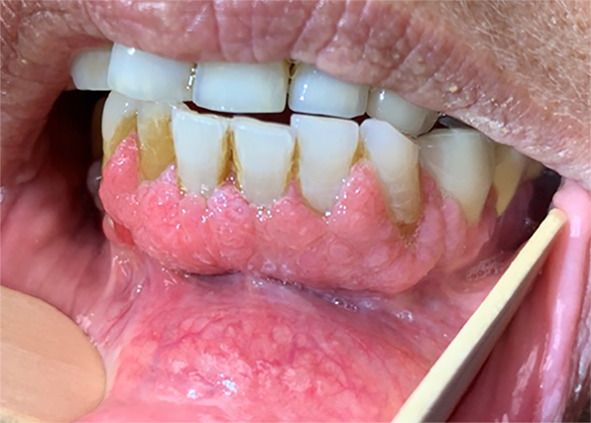
Verrucous hyperplasia was noted to predominantly involve bilateral mandibular alveolus and be centered in the symphysis and para-symphyseal regions. The area seemed somewhat textured throughout but homogenous without specific standout regions. (Patient 1)

**Fig. 2:**
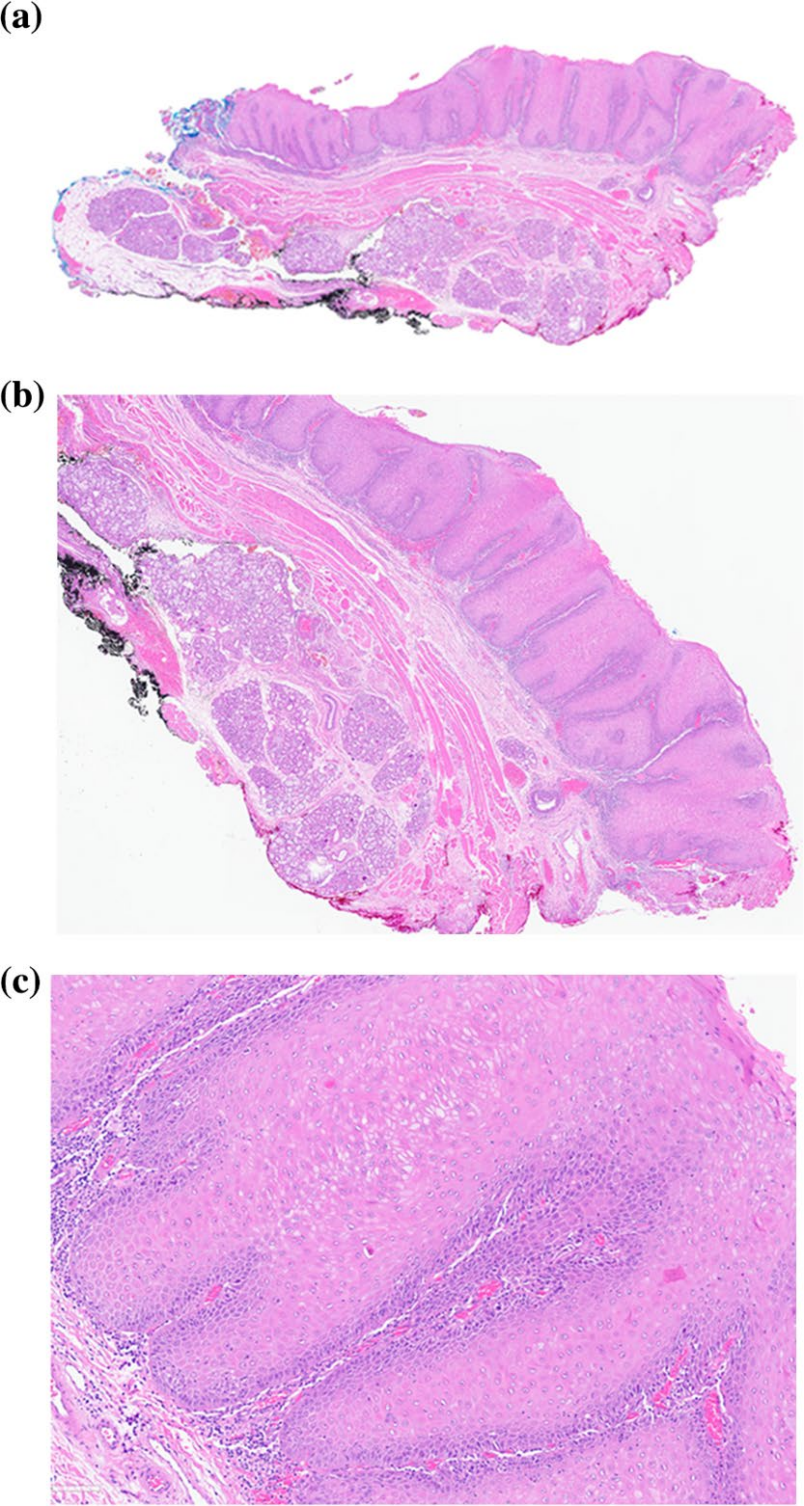
Histology shows proliferation with both exophytic and endophytic features, forming a bulky lesion with numerous tongue- or finger-like epithelium extending deeply and broadly in a pushing and sawtooth appearance into the underlying stroma. The cytologic atypia is present, however it is minimal to mild. H&E, 2 × 10 (a), 4 × 10 (b), and 10 × 10 (c). (Patient 2)

Figure 1 shows a representative clinical image of a typical verruciform, somewhat textured, and relatively homogenous leukoplakia, affecting the lower gum. On histology (Fig. 2), the squamous epithelium shows both exophytic and endophytic proliferative features, forming a bulky lesion with numerous tongue- or finger-like epithelial projections, focally coalescing, extending broadly in a pushing and sawtooth appearance into the underlying stroma with displacement and splaying bundles of fibrous connective tissue. The bulky tissue projected into the oral cavity lumen, but with broad rete rather than in a verrucous configuration, showing a much more rounded and cauliflower-like appearance. Hyperkeratosis and parakeratosis were present, but there were no church spire-type features or areas of parakeratotic crypting. Mitoses were not increased above the basal/parabasal layers. There was focal paradoxical maturation, with squamous eddies at the basal zone, associated with acute and chronic inflammatory cells. Significant pleomorphism was absent. The overall morphologic features of “barnaculate” carcinoma with a “stuck-on” appearance were present, lacking well developed features of either VC or CSCC. The other cases all demonstrated similar histologic features and thus are not further illustrated.

## Molecular Characterization

All molecular findings of *TERT* and *HRAS* oncogenic variants are listed in Table 2. Six specimens of BC from the four patients revealed alterations of *TERT* and *HRAS* genes. The *TERT* oncogenic variants presented in the promotor regions: two cases at c.-124C > T, one case at c.-57A > C, and one case at c.-146C > T; all six BC samples had the *HRAS* oncogenic variants (either c.34G > A, p.G12S or c.37G > T, p.G13C). Interestingly, two subsequent BC samples from a single patient (patient two) had different *HRAS* oncogenic variants.

**Table 2:** Summary of molecular findings of *tert* and *hras alterations*

Patient	Specimen code	Oral cavity location	Diagnosis		TERT alteration*s*	*HRAS* Alterations
1	1A	Right lower gum	**BC**		**c.-124C > T**^b^	**c.34G > A, p.G12S**^d^
	1B	Gingiva	**BC**		**c.-124C > T**^b^	**c.34G > A, p.G12S**^d^
2	2A	Right tongue	**BC**		**c.-124C > T**^b^	**c.37G > T, p.G13C**^d^
	2B	Right gum/mandible	CSCC, invasive		c.-124C > T^b^	Not detected^c^
	2C	Left upper lip	**BC**		**c.-124C > T**^a^	**c.34G > A, p.G12S**^a^
3	3A	Right tongue	**BC**		**c.-57A > C**^b^	**c.34G > A, p.G12S**^d^
	N/A	Right tongue (adjacent to BC)	Reactive squamous mucosa		Not detected^b^	Not tested
4	4A	Right lower lip	**BC**		**c.-146C > T**^b^	**c.37G > T, p.G13C**^d^
5	N/A	Mouth floor	CSCC, invasive		c.-124C > T^b^	Not detected^d^
6	N/A	Anterior tongue	CSCC, invasive		c.-146C > T^b^	Not detected^d^
	N/A	Tongue	CSCC, invasive		c.-146C > T^b^	Not detected^d^
	N/A	Left submandibular LN	CSCC, metastatic		c.-146C > T^b^	Not detected^d^
	N/A	Left neck level I LN	CSCC, metastatic		c.-146C > T^b^	Not detected^d^
7	N/A	Oral cavity	Non-cancerous PVL		Not detected^b^	Not detected^d^

Assays: a – NGS-based Analysis at Ashion; b – Sanger sequencing at COH; c – NGS-based analysis at Fulgent; d – NGS-based analysis at COH.

BC barnaculate carcinoma, CSCC conventional squamous cell carcinoma, LN lymph node, NGS next-generation Sequencing; COH City of Hope, PVL proliferative verrucous leukoplakia, N/A Not applicable.

Specimen Code: Corresponding to specimen codes shown in Table 1

Three non-cancerous PVL tissue specimens were tested. One specimen did not show either *TERT* promoter or *HRAS* alterations. However, the other two failed the tests due to poor sample quality. One squamous mucosal sample of right tongue immediately adjacent to a BC was analyzed with no *TERT* promoter alterations detected.

Six CSCC samples from three patients with primary oral lesions and lymph node metastases were examined. One of six samples came from a patient who had BC subsequently; the other patients did not have BC. All six samples revealed similar *TERT* promoter oncogenic variants, but did not have *HRAS* gene alterations.

For all the specimens tested, additional molecular findings of oncogenic variants other than *TERT* and *HRAS* alterations are summarized in Supplemental Table.

## Discussion

The diagnosis of oral epithelial dysplasia and SCC is often challenging and predominantly relies on evaluating various degrees of architectural and cytologic changes. Given the overlap of morphologic changes among reactive/reparative lesions and squamous carcinoma, significant inter- and intra-observer variability is seen [15].

PVL is a distinct oral precursor disorder, presenting over time with multifocal oral cavity leukoplakia [5, 15]. PVL frequently affects older females, mainly involving gingiva and peri-tooth insertion sites. The lesions typically demonstrate multifocal leukoplakia, with a papillomatous to verrucous appearance clinically, although variation is common. The histologic features are also diverse, but typically show a characteristic abrupt, corrugated orthohyperkeratotic proliferation that may show skip regions, progressing to bulky hyperkeratotic verruciform epithelial proliferations with expansion of rete and a combined exo- and endophytic growth. The disease usually follows a relentless clinical course with multiple lesions identified over time, with ultimate progression to cancer, whether BC or other subtypes of SCC in nearly all patients [5, 16]. In a recent review and meta-analysis by González-Moles, et al., PVL associated oral SCC showed a lower overall mortality rate than conventional oral SCC (21.3% vs 34.7–50%) [17].

BC is a specific subtype of well-differentiated SCC commonly identified within the clinical setting of PVL [5]. The term “barnaculate” is used to describe the epithelial proliferation with features similar to a barnacle stuck on a surface. BC usually shows features of exophytic and endophytic convoluted and complex epithelial proliferation with long and broad projections and a pushing border into the subepithelial tissue. There is often surface hyper-, ortho- and parakeratosis. There is frequently an acute inflammatory infiltrate, especially at the base. There is no significant pleomorphism, with limited mitoses present. Paradoxical maturation may be seen. These features were well represented in our cases (Fig. 2), which lacked the typical exophytic verruciform/church spire-type or papillary features of VC or papillary SCC. One distinctive feature of BC is a generally rounded luminal surface without pleomorphism, while lacking the shearing or masticatory surface effects that can be seen in verrucous carcinoma. Overall, these lesions tend not to be associated with metastatic disease to the lymph nodes. It is difficult in the setting of PVL to know if there is a new lesion developing at the same or immediately adjacent site, versus a true local recurrence, and as such, there seems to be a more indolent clinical course for BC, but further studies are necessary to confirm this finding. Due to very limited pleomorphism, BC may be misinterpreted as a benign or reactive disorder, especially when there is significant inflammatory infiltrate and/or the presence of a concurrent fungal infection (candida species). It is important to state that BC is a morphologically distinct pattern of features, recognizing that the overlap with reactive lesions and other SCC subtypes is present. It would be prudent to make certain a biopsy of adequate size and depth to include the underlying stroma is included in order to make assessment meaningful. Bulky, atypical epithelial proliferations with elongated rete may mimic BC, although in general the “stuck-on” appearance where the epithelium is at the same level as the adjacent epithelium without significant pushing extension is a helpful finding. As such, it is possible that a BC may be present in a patient not yet diagnosed with PVL, although this is a theoretical consideration as the subtype of carcinoma is only recently introduced without large randomized or controlled studies performed to see if the tumor may be present in non-PVL patients. Certainly, both BC and CSCC can occur in the setting of PVL. In our study, CSCC has been found to occur even between two events of BCs.

Due to these practical difficulties of diagnosing BC morphologically, molecular findings characteristic for BC may be helpful, specifically for the differential diagnosis between noncancerous PVL lesions and BC. In this study, six specimens from four BC patients demonstrated oncogenic variants in *TERT* promoter regions and *HRAS* genes. The findings of two subsequent BC specimens in one patient carrying two different *HRAS* oncogenic variants may indicate two unrelated sporadic processes of BC development. Interestingly, all six samples of primary invasive CSCC and lymph node metastases from three different patients, including one sample from the patient who developed CSCC between two BC events, demonstrated similar *TERT* gene promotor mutations as seen in BC, but lacked *HRAS* gene alterations. Further, one noncancerous PVL sample in our study revealed neither *TERT* promoter nor *HRAS* variants. *TERT* promoter alterations were not detected in one unaffected tongue mucosal sample from a patient who had an adjacent BC.

Oncogenic variants in *TERT* promoter, especially -124C > T, have been well documented in various types of cancers, including HNSCC. The C-to-T mutation occurs within the GC-rich promoter area of the *TERT* gene. The variant creates a de novo binding site for the E26 (ETS) transcription factor, causing increased gene expression. The variants of -57A > C and -146C > T have also been seen in various cancer types, such as melanoma [18]. *HRAS* is a proto-oncogene and its product has GTPase activity and is involved in signal transduction, with the downstream *RAF* and *PI3K*, to promote cellular proliferation and survival [11]. The hotspot missense mutation of G12 is associated with significant reduction of the GTPase activity and defective GTP hydrolysis, leading to constitutively activated signal transduction [19, 20]. *HRAS* oncogenic variants have been documented in 4–9% of HNSCCs [21], although not seen in any CSCC samples in this study.

This study has shown both *TERT* promoter and *HRAS* oncogenic variants in all six BC specimens from four different patients, but not in benign oral mucosal, non-cancerous PVL lesions or CSCC samples tested, suggesting these recurrent genomic changes are probably common or potentially even characteristic in BC. While possibly unnecessary, it is potentially helpful for diagnosis in difficult cases with differential diagnosis between BC and noncancerous PVL lesions or other reactive/reparative lesions.

The co-occurrence of *TERT* and *HRAS* oncogenic variants is low in HNSCCs with less than 2% calculated. The morphological and clinical features of these tumors with co-occurring *TERT* and *HRAS* oncogenic variants were not mentioned in the study reported by Colemen, et al. [13]. It would be interesting to know whether at least a few of these tumors are indeed BCs.

We demonstrated one PVL specimen to have no *TERT* or *HRAS* oncogenic variants detected. Certainly, more studies to examine the status of these gene alterations in noncancerous PVL specimens are necessary. However, in the future, if these specimens were found to carry such cancer-associated *TERT* and *HRAS* oncogenic variants, the diagnosis of noncancerous PVL for the tested samples should perhaps be re-considered.

For all the tumor specimens tested, in addition to *TERT* and *HRAS* alterations, several other oncogenic variants have also been found (see Supplemental Table). Due to the limitation of specimen number of the study, further investigation is needed to understand the significance of this molecular information obtained.

Our conclusions are preliminary due to limited case numbers. Further extensive studies are needed to validate the findings. The tumorigenic roles of these oncogenic variants, especially in the process of evolving from a precancerous state, such as PVL to SCC, have not yet been identified. The clinical significance in terms of disease prognosis is also uncertain. Additional studies may elucidate the nature of this unique disease and cancer subtype.

In summary, our studies have demonstrated oncogenic variants in the promoter region of *TERT* and *HRAS* genes in all tested specimens of BC. The identification of these variants may be helpful in the diagnosis of difficult or challenging cases of this category.

## Supplementary Information

Below is the link to the electronic supplementary material.

Supplementary Material 1

## Data Availability

Availability of data and material is possible upon reasonable request, deidentified for maintenance of anonymity and compliance with IRB approval.
