# Supplementary material for: Barnaculate Carcinoma in Four Patients: Verrucoid Squamous Cell Carcinoma Subtype with TERT and HRAS Oncogenic Variants
Source: Head Neck Pathol. 2026 Jan 5;20(1):6. doi: 10.1007/s12105-025-01870-3 (PMC12770093; doi:10.1007/s12105-025-01870-3)
Supplement: Supplementary file 1 — Supplementary Material 1 [file 12105_2025_1870_MOESM1_ESM.docx]

| **SUPPLEMENTAL TABLE. SUMMARY OF MOLECULAR FINDINGS OF GENE ONCOGENIC VARIANTS OTHER THAN *TERT* AND *HRAS* ALTERATIONS** | | | | | | | | | |
| --- | --- | --- | --- | --- | --- | --- | --- | --- | --- |
|  | | | | | | | | | |
| **Patient** | **Specimen Code** | | **Oral Cavity Location** | **Diagnosis** | | **Other Gene Oncogenic Variants** | | | |
| 1 | 1A | | Right lower gum | **BC** | | Not detected1^1^ | | | |
|  |  | |  |  | |  | | | |
|  | 1B | | Gingiva | **BC** | | ***B2M*** (c.21dup, p.A8Sfs*49)^3^ | | | |
|  |  | |  |  | |  | | | |
| 2 | 2A | | Right tongue | **BC** | | ***NOTCH1*** (c.4357G>T, p.E1453*); ***PIK3CA*** (c.1636C>A, p.Q546K)^1^ | | | |
|  |  | |  |  | |  | | | |
|  | 2B | | Right gum/mandible | CSCC, invasive | | ***ARID5B*** (c.733+1G>A); ***CDKN2A*** (c.191_210del20, p.L64Qfs*49; ***FAT1*** (c.10679C>G, p.S5360*); ***FAT1*** (c.7040_7050del11, p.T2347Rfs*14); ***FBXW7*** (c.1435G>A, p.G479R); ***NFE2L2*** (c.92G>A, p.G31E); ***NOTCH1*** (c.151dupG, p.A51Gfs*92); ***NOTCH1*** (c.4357G>T, p.E1453*)^5^ | | | |
|  |  | |  |  | |  | | | |
|  | 3B | | Left upper lip | **BC** | | ***CDKN2A*** (L64fs); ***EP300*** (Q821*); ***PIK3CA*** (E542K)^2^ | | | |
|  |  | |  |  | |  | | | |
| 3 | 3A | | Right tongue | **BC** | | ***NOTCH1*** (c.3249_3259delinsG, p.C1083Wfs*93); ***TP53*** (c.796G>A, p.G266R)^4^ | | | |
|  |  | |  |  | |  | | | |
|  | 3A | | Right tongue (adjacent to BC) | Reactive squamous mucosa | | Not tested | | | |
|  |  | |  |  | |  | | | |
| 4 | 4A | | Right lower lip | **BC** | | ***CTCF*** (c.1506C>G, p.Y502*); ***FAT1*** (c.5630dup, p.V1878Cfs*8); ***IGF2*** (AMP); ***SLIT2*** (c.2277-1G>T)^3^ | | | |
|  |  | |  |  | |  | | | |
| 5 | N/A | | Mouth floor | CSCC, invasive | | ***CDKN2A*** (c.458-2_458-1insAT); ***IGF2*** (AMP); ***TP53*** (c.817C>T, p.R273C) ^3^ | | | |
|  |  | |  |  | |  | | | |
| 6 | N/A | | Anterior tongue | CSCC, invasive | | ***FBXW7*** (c.1393C>T, p.R465C); ***PIK3CA*** (c.1258T>C, p.C420R); ***TP53*** (c.423_427del, p.P142Afs*5) ^3^ | | | |
|  |  | |  |  | |  | | | |
|  | N/A | | Tongue | CSCC, invasive | | ***ASXL1*** (c.2393_2397dup, p.A800fs*8); ***CCND1*** (AMP); FGF3 (AMP); ***FGF4*** (AMP); FGF19 (AMP); ***FBXW7*** (c.1393C>T, p.R465C); ***PIK3CA*** (c.1258T>C, p.C420R); ***TP53*** (c.423_427del, p.P142fs*5)^3^ | | | |
|  |  | |  |  | |  | | | |
|  | N/A | | Left submandibular LN | CSCC, metastatic | | ***ASXL1*** (c.2576_2580dup, p.A861fs*8); ***CCND1*** (AMP); ***FBXW7*** (c.1393C>T, p.R465C); ***FGF3*** (AMP); ***FGF4*** (AMP); ***FGF19*** (AMP); ***PIK3CA*** (c.1258T>C, p.C420R); ***TP53*** (c.423_427del, p.P142fs*5)^3^ | | | |
|  | N/A | | Left neck level I LN | CSCC, metastatic | | ***ASXL1*** (c.2393_2397dup, p.A800fs*8); ***CCND1*** (AMP); ***FGF3*** (AMP); ***FGF4*** (AMP); ***FGF19*** (AMP); ***ERCC1*** (AMP); ***ERCC2*** (AMP); ***FBXW7*** (c.1393C>T, p.R465C); ***PIK3CA*** (c.1258T>C, p.C420R); *TP53* (c.423_427del, p.P142fs*5)^3^ | | | |
| 7 | N/A | | Oral cavity | Non-cancerous PVL | | Not tested | | | |
|  |  | |  |  | |  | | | |
|  |  |  | | |  | |  |  |  |

**Assays:** 1: CMDL HopeSeq Solid Tumor panel (including 87 gene mutation analysis); 2: CMDL HopeSeq Solid Tumor Panel (including 89 gene mutation analysis); 3: CMDL HopeSeq Solid Tumor Panel (including 523 gene entire exon analysis); 4: Ashion GEM Extra^®^; 5: Fulgent The Lumera NGS Profile of Solid Tumor.

**Abbreviations:** BC - barnaculate carcinoma; CSCC – conventional squamous cell carcinoma; LN – lymph node; PVL: proliferative verrucous leukoplakia; CMDL: Clinical Molecular Diagnostic Laboratory; N/A – Not applicable.

**Specimen Code:** Corresponding to specimen codes shown in Table 1
